# BUILDING THE OLDER ADULT BEHAVIORAL HEALTH WORKFORCE: OREGON’S CENTER OF EXCELLENCE FOR BEHAVIORAL HEALTH & AGING

**DOI:** 10.1093/geroni/igae098.4136

**Published:** 2024-12-31

**Authors:** Leah Brandis, Walter Dawson, Allyson Stodola, Dani Himes, Nirmala Dhar, Lindsey Smith, Paula Carder

**Affiliations:** OHSU, Portland, Oregon, United States; OHSU, Portland, Oregon, United States; Portland State University, Portland, Oregon, United States; Portland State University, Portland, Oregon, United States; Oregon Health Authority, Portland, Oregon, United States; OHSU-PSU School of Public Health, Portland, Oregon, United States; OHSU-PSU School of Public Health, Portland, Oregon, United States

## Abstract

The need for behavioral health workforce support is prevalent nationwide. Older adult Oregonians have a 26% prevalence of anxiety disorders, 12% prevalence of serious mental illness and Oregon has the 4th highest rate in the nation for older adult deaths due to intentional self-harm. The newly established Oregon Center of Excellence for Behavioral & Aging (OCEBHA) is designed to support the behavioral health needs of older adults. A primary objective is to boost the capacity of the workforce to support older adults living with behavioral health needs. The workforce is defined broadly and includes behavioral health and aging subject matter experts, clinical and administrative health care workers, direct care workers in long-term care settings, first responders, care managers, and older adults themselves. There are already workforce training efforts happening across Oregon, therefore, in order to collaborate and align efforts, OCEBHA has launched a coalition of organizations (n= 15) who already train this workforce. This coalition will meet quarterly to share upcoming training opportunities in order to support the sectors’ efforts, identify gaps, as well as brainstorm future collaborations. In addition, Oregon has recently received the state’s first Geriatric Workforce Enhancement Program (GWEP) award and OCEBHA is already working closely with the Oregon GWEP to collaborate on future training endeavors. Based on identified gaps, a comprehensive workforce training plan for the next four years is in development. The intention is to collaborate with partners across the state to ensure training is available to meet basic older adult behavioral health competencies.

